# Effect of patient age awareness on diagnostic agreement of chronic or aggressive periodontitis between clinicians; a pilot study

**DOI:** 10.1186/s12903-016-0258-0

**Published:** 2016-07-25

**Authors:** Sarah Oshman, Edgard El Chaar, Yoonjung Nicole Lee, Steven Engebretson

**Affiliations:** New York University College of Dentistry, Advanced Education Program in Periodontics, 345 East 24th Street, New York, NY 10016 USA

**Keywords:** Aggressive periodontitis, Periodontal diagnosis, Chronic periodontitis, Periodontal disease

## Abstract

**Background:**

The aim of this pilot study was to test whether diagnostic agreement of aggressive and chronic periodontitis amongst Board Certified Periodontists, is influenced by knowledge of a patient’s age. In 1999 at the International World Workshop age was removed as a diagnostic criteria for aggressive periodontitis. The impact of this change on the diagnostic reliability amongst clinicians has not yet been assessed.

**Methods:**

Nine periodontal case reports were twice presented to sixteen board certified periodontists, once with age withheld and again with patient age provided. Participants were instructed to choose a diagnosis of Chronic Periodontitis or Aggressive Periodontitis. Diagnostic agreement was calculated using the Fleiss Kappa test.

**Results:**

Including the patients’ age in case report information increased diagnostic agreement (the kappa statistic) from 0.49 (moderate agreement) to 0.61 (substantial agreement).

**Conclusion:**

These results suggest that knowledge of a patients’ age influenced clinical diagnosis, when distinguishing between aggressive periodontitis and chronic periodontitis, which may in turn impact treatment decision-making.

## Background

Over the years researchers and clinicians have used many different terms to describe periodontal destruction in the younger population including; periodontosis, juvenile periodontitis, early onset periodontitis, and aggressive periodontitis. Classification systems for the periodontal diseases are updated periodically according to clinical and scientific advances in the literature to aid clinicians in the study, diagnosis, and treatment of the periodontal diseases [1, 2]. The most recent update to the classification system for the periodontal diseases was at the 1999 International World Workshop, where scientists and clinicians met to update and rewrite the 1989 guidelines. Key modifications to the 1989 guidelines included reclassifying “adult periodontitis” as “chronic periodontitis” and “early­onset periodontitis” as “aggressive periodontitis.” Within the 1989 classification system, age at onset was a criteria for diagnosis of adult periodontitis, early onset periodontitis, pre-pubertal periodontitis, and juvenile periodontitis. An age of 30 years was used to distinguish between juvenile or Aggressive Periodontitis and Adult or Chronic Periodontitis in the 1989 classification system [3–5]. However, in 1999 the age criteria that had previously distinguished aggressive and chronic periodontal disease was removed, leaving diagnosis of these forms of periodontitis to be based solely on clinical features such as; rate of disease progression; amount of periodontal destruction relative to local factors such as plaque and calculus; and a specific disease pattern of greater destruction surrounding first molars and central incisors [6–8]. The use of microbiologic testing as a means to distinguish between the two forms of disease has been explored but currently microbial findings have not been shown to aid diagnostic capability [4, 9].

The substantial overlap of clinical features of aggressive and chronic periodontitis can create a diagnostic challenge that could lead to disagreement amongst clinicians and researchers concerning diagnosis. Misclassification of these diseases may influence the outcome of epidemiologic studies, and clinical decision-making. A recent task force report by the American Academy of Periodontology confirmed the importance of the diagnosis of chronic or aggressive periodontitis and the implications of diagnosis related to treatment, prognosis, and referral of patients with disease [10]. This report also announced that the American Academy of Periodontology will begin an update to the 1999 classification for periodontal disease in 2017 and will address specific areas of concern such as the diagnosis of chronic verses aggressive periodontitis. The task force cited patient age as a general consideration when distinguishing between chronic and aggressive periodontitis. However, the impact of utilizing patient age as a diagnostic criteria on diagnostic agreement amongst clinicians has not been extensively studied [10]. The aim of this pilot study was to determine whether withholding a patient’s age from a case report influenced the diagnostic agreement, of chronic or aggressive periodontitis, amongst Board Certified Periodontists.

## Methods

### Study design and populations

**Fig. 1:**
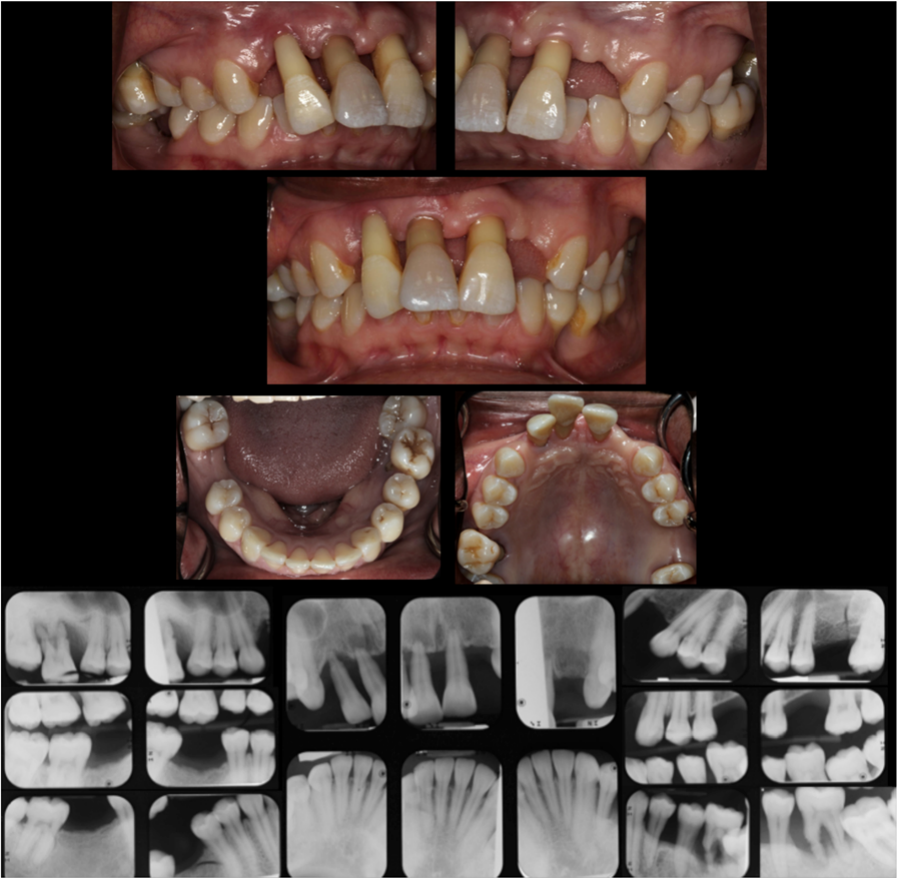
The included figure is sample of a full set of radiographs and intraoral photos provided in the electronic case reports. This particular set is Case 8; A 27 year old female who presented to the NYUCD Periodontics clinic with no significant medical or social history and a chief complaint of spaces between her front teeth

Nine patients with moderate to severe periodontal attachment loss were selected from the patient population of the Advanced Education Program in Periodontics at the Ashman Department of Periodontology and Implant Dentistry at the New York University College of Dentistry (NYUCD) between 2010 and 2014. Exclusion criteria were; periodontitis as a result of systemic disease, patients with diabetes mellitus, patients with mixed dentition, patients with implants, and patients with extensive restorative work. Patients with mixed dentitions, extensive restorations, or implants were excluded as the appearance of these in a clinical exam could provide clues to a patient’s age. Case reports were prepared based on the comprehensive periodontal evaluations preformed by the graduate students enrolled in the Advanced Education Program in Periodontics. These case reports included a full set of radiographs and periodontal charting with the following clinical periodontal measures; pocket depth, bleeding on probing, clinical attachment loss, mobility, and furcation involvements. Measurements of pocket depth, clinical attachment loss, and bleeding on probing were recorded for six surfaces of all teeth; mesiobucal, the direct buccal, distobuccal, mesiolingual, direct lingual, and distolingual [11]. The Hamp classification was used when assessing furcation involvement and the Miller classification was used to record tooth mobility [12, 13]. The patients’ chief complaint, medical history and dental history were included in the case report and care was taken to remove all personal identifiers. A sample of radiographs and intraoral photos from case report 8 is available as Fig. 1. Case reports were de-identified and protected health information was not used in accordance with the Health Portability and Accountability Act privacy rule. Patients gave written informed consent for the use of their de-identified information for research purposes. This study protocol was evaluated by the International Review Board Human Research Protection Program, NYU School of Medicine, NYU Langone Medical Center, using the approved Self-Certification Form, on file with NYUCD Advanced Education Program in Periodontics, and was deemed exempt from IRB review.

The board certified periodontal faculty at NYUCD were asked to participate as examiners for this pilot study and sixteen members agreed. The faculty included both full and part time members, and each was a Diplomat of the American Board of Periodontology. The board certified periodontists who participated in this study were informed of the nature of the study, and all gave written informed consent and were provided the ability to opt out without consequence.

### Data collection and analysis

The cases were presented twice to the sixteen examining periodontists via electronic format. The case reports consisted of a complete periodontal chart, a case narrative, intraoral photos, and a complete radiographic series. The examining periodontists were asked to evaluate each case independently, without consultation, and provide a diagnosis. For each case, based only on the information provided, the periodontist was prompted to choose either a diagnosis of chronic or aggressive periodontitis. The examining periodontists were first presented with the case reports without the patient’s age. The second time the same cases were presented with the patient’s age included. The examining peridontisits were not permitted to see or change their diagnostic selections from the first set of case presentations while reviewing the cases the second time. None of the examiners were directly involved in supervising the graduate students during data collection for these cases. The diagnostic agreement among the examiners was compared for each round using Fleiss’ Kappa statistic and the Landis and Koch’s 1977 interpretation of Kappa Values [14, 15].

## Results

Table 1 shows the number of examining periodontists who selected chronic or aggressive periodontitis for each case when the patient’s age was withheld from case information and again when the patient’s age was included. In the first instance when patient’s age was excluded from the case information the Kappa value was 0.494, which according to Landis and Koch can be interpreted as ‘moderate’ agreement [14]. In the second instance when the patient’s age was included the Kappa statistic was 0.611, which can be interpreted as ‘substantial’ agreement [14]. The change in diagnostic agreement from 0.494 to 0.611 represents an improvement in agreement from ‘moderate’ to ‘substantial’ when patient’s age was included in the case report information. All collected data sets have been reported and made available in Table 1.

**Table 1:** Number of examiners that chose a diagnosis of aggressive or chronic periodontitis for each case report

Case	Patient’s Age Excluded From Case Information		Patient’s Age Given with Case Information	
	Raters who Selected Aggressive Periodontitis	Raters who Selected Chronic Periodontitis	Raters who Selected Aggressive Periodontitis	Raters who Selected Chronic Periodontitis
1	15	1	16	0
2	16	0	16	0
3	0	16	1	15
4	3	13	0	16
5	2	14	9	7
6	10	6	11	5
7	7	9	15	1
8	8	8	11	5
9	0	16	0	16
Kappa	0.494; Moderate Agreement		0.611; Substantial Agreement	

The number of Board Certified Periodontists that chose a diagnosis of Aggressive or Chronic periodontitis was recorded for each case report. The cases were first presented with age withheld from case information and then a second time with age included. The agreement between examining periodontists diagnoses was calculated using the Kappa statistic and interpreted following Landis and Koch [11, 12]

## Discussion

This pilot study demonstrated that inclusion of a patient’s age in the case information significantly improved diagnostic agreement among 16 board certified examining periodontists when asked to distinguish between chronic and aggressive periodontitis. The level of agreement between clinicians was compared overall between instances when the patient age was withheld and when it was given. Kappa was calculated for both of theses scenarios and there was an increase in inter-examiner agreement from moderate to substantial when age was provided. Also when observing the results for a given cases report, while not as statically significant as a kappa calculation, one can note that examiners did select a different diagnosis for the same patient given age (Table 1). For example in case four, when age was excluded, four examiners chose a diagnosis of aggressive periodontitis but once given the patient’s age for that case, all examiners selected chronic periodontitis. Even though this one scenario is not statistically significant, it does bring to light the influence patient age can have on clinicians’ diagnoses and presents the need for further study into the influence of diagnostic criteria on reliability and clinician agreement.

In this pilot study, Fleiss Kappa statistic was used as a means to compare the degree of diagnostic agreement over that, which would be expected by chance. We interpret these results to be clinically significant as treatment planning and clinical decision-making will likely be influenced. To our knowledge this is the first pilot study to demonstrate that patient age influences diagnostic agreement amongst clinicians. The results of this pilot study suggest that patient age may be a significant factor in clinical diagnosis and calls into question whether patient age should remain excluded from the diagnostic criteria for aggressive periodontitis. Since a high degree of diagnostic agreement amongst clinicians is desirable, a revision of clinical criteria to distinguish between aggressive and chronic periodontitis should be considered. Larger study populations of both cases and examiners and a study design that includes variables other than age, could help determine which specific criteria of disease classification specifically increase inter-examiner reliability. Our results are in agreement with the American Academy of Periodontology’s recent task force report, which suggested that patient age should be a consideration when diagnosing chronic or aggressive periodontitis and that a revision to the diagnostic criteria that distinguishes the two forms of the disease should be considered. The desire for a re-evaluation of our diagnostic system is also in line with a recent publication in reaction the previous mentioned task force report that calls for a profound reconsideration of what is hoped to be achieved with periodontal classification [16].

## Conclusion

This pilot study demonstrates a need for further investigation of both the effect of patients’ age in the diagnosis of aggressive verses chronic periodontitis and the need for consideration of a patient’s age during the update to our current classification. Reliable and reproducible diagnostic criteria are necessary for communication between clinicians, implementation of standardized care and accuracy of epidemiological studies. Larger studies with greater population sizes of both examiners and case reports should be conducted to examine the full extent of the influence of specific diagnostic criteria, such as patient age, on the diagnostic agreement amongst clinicians for both aggressive and chronic periodontitis.

## Abbreviations

NYUCD, New York University College of Dentistry
